# Genetic Insights into the Economic Toll of Cell Line Misidentification: A Comprehensive Review

**DOI:** 10.3390/medsci14010025

**Published:** 2026-01-05

**Authors:** Ralf Weiskirchen

**Affiliations:** Institute of Molecular Pathobiochemistry, Experimental Gene Therapy and Clinical Chemistry (IFMPEGKC), RWTH University Hospital Aachen, D-52074 Aachen, Germany; rweiskirchen@ukaachen.de; Tel.: +49-(0)241-8088683

**Keywords:** STR profiling, ICLAC, Cellosaurus, CLASTR, cell culture, cell lines, cell misidentification, ANSI/ATCC standards

## Abstract

Cell line misidentification, first exposed when HeLa cells were shown to contaminate dozens of “unique” cultures, now compromises roughly one in five lines and renders thousands of papers potentially unreliable, propagating unreliable data through hundreds of thousands of citations. The financial fallout is vast with irreproducible research linked to faulty cell stocks costing the United States an estimated $28 billion each year. Today, authentication is rapid, cheap and highly accurate. Modern 24-plex short tandem repeat (STR) kits, analyzed by six-dye capillary electrophoresis and benchmarked against public databases, verify a culture in half a day for less than €40, lowering the probability of mistaken identity to less than 10–15. Complementary SNP panels, low-pass genome sequencing, digital PCR and nascent methylation “age clocks” close remaining blind spots such as aneuploidy or mixed-species co-cultures. Monte-Carlo modeling shows that even at a contamination risk of 0.07% routine STR testing yields a five-year return on investment above 3000% for a mid-size lab. Reflecting this evidence, ANSI/ATCC standards, NIH and Horizon Europe grants, major journals and FDA/EMA guidelines now encourage, recommend, or make authentication mandatory. This review discusses the historical roots and economic losses resulting from cell misidentification and contamination and offers a pragmatic roadmap to prevent working with falsified cell lines. It is further discussed that FAIR-compliant data archiving and integration of STR workflows into laboratory data management systems will allow laboratories to shift from sporadic testing of cell quality to continuous, artificial intelligence-supported assessments.

## 1. Introduction

**Figure 1:**
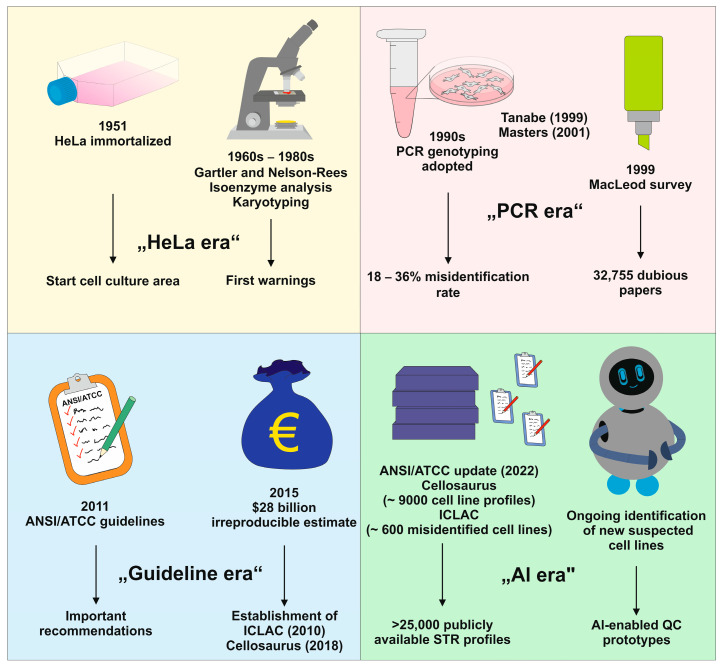
Seventy-year timeline of key events shaping cell line authentication. Milestones include the following four eras: initial HeLa contamination episode, the introduction of PCR-based identification, regulatory guidelines, and the current transition toward AI-assisted, blockchain-secured provenance.

The modern age of cell culture began with the immortalization of HeLa cells in 1951. The “HeLa era” unlocked unprecedented experimental freedom but also planted the seeds of a problem that would plague biology for decades (Figure 1).

The cell line was established by continuous serial culture passage at John Hopkins Hospital in Baltimore, Maryland, using cervical cancer cells taken on 8 February 1951, from the 31-year-old African American woman Henrietta Lacks [1,2]. In the early years, cell stocks were transferred informally from lab to lab, often in thermos flasks wrapped in newspaper, without standardized nomenclature or quarantine procedures. In 1967, Stanley Gartler published data showing that HeLa had infected and taken over the cultures of 18 different cell lines [3]. Later, cytogeneticist Walter Anthony Nelson-Rees (11 January 1929–23 January 2009) began systematically developing methods and karyotyping common cell lines. He discovered, through analyzing type A mobility for glucose-6-phosphate dehydrogenase and the lack of a Y chromosome, that HeLa cells had already contaminated over 40 supposedly “unique” cultures, including those labeled KB (CVCL_0372), Chang liver (CVCL_0238), HEp-2 (CVCL_1906), HBT3 (CVCL_D281), and Intestine 407 (INT407, CVCL_1907) [4,5]. Both Gartler and Nelson-Rees were met with a mixture of disbelief and resentment [6,7]. Moreover, in 1981 Nelson-Rees was accused by *Nature* of vigilantism [8], and many of the researchers who had generated imposter cell lines did not seem to learn the lessons of cross-contamination of their own cell lines from evidence that these two scientists presented.

The issue remained largely anecdotal until the mid-1990s. In the “PCR era”, DNA fingerprinting methods revealed that contamination was not a sporadic annoyance but a systemic threat. An informal survey by MacLeod and colleagues in 1999 placed the misidentification rate of human tumor cell lines at 18% (45 out of 252) among commonly used lines, figures that shocked even seasoned researchers [9,10]. Work by van Bokhoven and colleagues found that about 33% of prostate cell lines were misidentified [11,12]. Similarly, Schweppe and colleagues showed that nearly 50% of thyroid cell lines were misidentified [13].

Two developments then propelled the problem into the spotlight: the genomics revolution, which linked millions of dollars in sequencing investments to the authenticity of source material, and the emergence of online bibliometrics, which allowed scholars to quantify how errors propagate through the citation network. A single misidentified line such as MDA-MB-435 (long thought to be breast cancer but genetically indistinguishable from the melanoma line M14), spawned more than 1200 publications between 1982 and 2016, many of which were used in pre-clinical research [14].

The economic impact of irreproducible research, of which cell-line problems constitute a major share, has been estimated at $28 billion per year in the United States alone [15]. The hidden costs are equally daunting: grant reviewers grow skeptical, investors become wary of translational science, and public trust erodes when high-profile papers are retracted. Stern and colleagues discussed the general financial costs of retracted articles that were funded by the NIH and calculated that each retracted articles accounted for a mean of $392,582 ± $423,256 [16].

Indeed, retractions stemming from cell line contaminations often make headlines precisely because they appear so avoidable [17]. The 2013 retraction note by the Radovanovic group in *Nature Methods* referring to a paper that they had published before, prompted by HEK-293 cross-contamination, invalidated three years of work and forced funders to reallocate resources mid-cycle.

Meta-researchers have searched PubMed and Web of Science to evaluate the extent of the issue. Horbach and Halffman found 32,755 papers referencing dubious and mislabeled cells with questionable data [18]. In 2021, Korch and Capes-Davis exemplary showed how widespread this problem is. They identified 8497 and 1397 published articles that used the HeLa derivatives HEp-2 (CVCL_1906) and Intestine 407 (CVCL_1907) extensively describing them as laryngeal cancer and normal intestine cells [19]. An update in 2024 discussed how non-verifiable cell lines could lead to the creation of new cell lines without published description of how cell lines were established, STR profile, or making cell lines available through external suppliers, a phenomenon known as “miscelling” [20].

**Figure 2:**
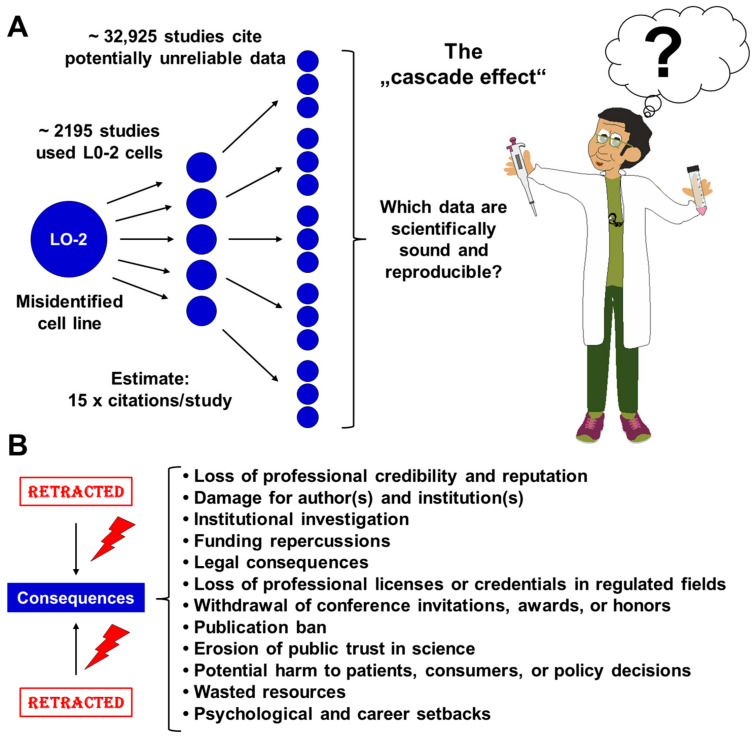
Consequences of cell line misidentification. (**A**) The cell line L-02, originally presented in 2013 as a human fetal hepatocyte cell line [21,23], has been used in approximately 2195 studies. Assuming each study was cited 15 times, around 32,925 instances of potentially unreliable data have been spread using this misidentified cell line. (**B**) The retraction of a paper containing invalid results can have various implications, including loss of professional credibility and reputation damage for the author(s) and their institution, institutional investigations (such as disciplinary measures like suspension or dismissal), funding repercussions (such as termination of ongoing grants, ineligibility for future funding, and repayment of misused funds), legal consequences (if potentially invalid results influenced clinical trials, product safety, or regulatory filings), potential loss of professional licenses or credentials in regulated fields (such as medicine or engineering), withdrawal of conference invitations, keynote talks, awards, or honors previously granted, difficulty publishing future work (as journals may impose bans or heightened scrutiny), erosion of public trust in science and potential harm to patients, consumers, or policy decisions. These factors are also associated with wasted resources (time, money, and effort spent by other researchers attempting to build on or replicate findings) and psychological and career setbacks for students and junior researchers involved in or associated with the work. However, unless intentional misconduct is documented, the issue is one of unintended error and compromised validity, not deliberate falsification.

Citation-tracking reveals a “cascade effect”, best illustrated by the example of the cell line L-02. Originally presented as a human fetal hepatocyte cell line [21], L-02 has been used in liver research in approximately 2195 studies [22]. Horbach and Halffman estimated that 32,755 original flawed papers resulted in about 500,000 citations, implying a mean of roughly 15 citations per irreproducible paper [18]. Applying this average to L-02 suggests on the order of 2195 × 15 ≈ 32,925 downstream citations to work relying on this misidentified line (Figure 2A). These calculations are intended to illustrate the potential scale at which potentially unreliable or invalid results can propagate through the literature due to unrecognized cell line misidentification, rather than to suggest deliberate fabrication or misconduct by the authors of the affected publications.

The implications of such misidentification are significant. Retracting any paper containing potentially unreliable data can have severe consequences for the authors and their institutions. The consequences may include a loss of professional credibility, potential institutional investigations, funding repercussions, and even legal consequences if the unreliable results impact clinical trials or regulatory filings. Additionally, researchers may encounter challenges in publishing future work due to increased scrutiny from journals, ultimately eroding public trust in science and potentially harming patients or consumers who rely on accurate data (Figure 2B).

**Figure 3:**
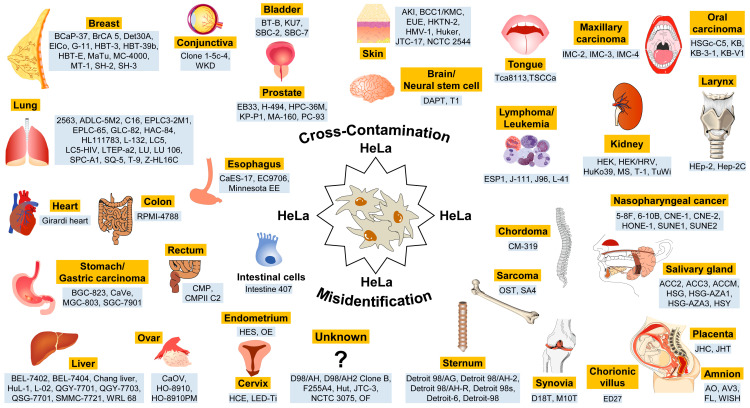
HeLa-contaminated cell lines originally reported to derive from diverse organs. This schematic provides an overview of widely used continuous cell lines that were once believed to originate from different organs. Embedded labels indicate the misidentified line(s). The data of HeLa-contaminated cell lines was taken from the [25].

Even worse: In the latest version of the Register of Misidentified Cell Lines, which is regularly updated by the International Cell Line Authentication Committee, there are approximately 150 cell lines listed that have been contaminated with HeLa cells [24]. These cell lines were originally believed to originate from various organs (Figure 3).

It is difficult to determine the extent of inaccurate data produced by these HeLa-contaminated cell lines, especially in terms of how frequently this incorrect information has been referenced and spread within the scientific community. The widespread use of misidentified cell lines not only compromises the credibility of individual studies, but can also result in significant harm, such as erroneous conclusions in clinical applications. There are so many examples of wasted work resulting from poor experimental design that there is an urgent need to adapt the dysfunctional biomedical system [26,27].

Given this backdrop, STR-based cell line authentication does not merely serve individual laboratories. It functions as a macro-economic stabilizer for the entire biomedical ecosystem and will prevent needless problems [28]. It is obvious that when scientists use misidentified cell lines, unreliable and irreproducible results will be established, leading to increased costs and misguided future studies [28]. In short, the history of cell line misidentification teaches an unambiguous lesson: unchecked error perpetuates cost escalation, leading to a decline in public confidence. Authentication, once viewed as an optional courtesy, has become a fiscal imperative, with a 25% reduction in the use of misidentified cell lines estimated to rescue billions of US$ in funding [29].

## 2. Evolution and Technical Nuances of Short Tandem Repeat Profiling

### 2.1. Short Tandem Repeat Profiling: Review Methodology and Literature Research Strategy

Given the broad relevance of cell misidentification, this article was designed as a narrative review informed by a structured, but non-systematic, literature search rather than a formally registered systematic review. During manuscript preparation, PubMed/MEDLINE, Web of Science Core Collection, and Scopus were searched using combinations of controlled vocabulary and free-text terms related to cell lines, misidentification, authentication technologies, reproducibility, and economic impact (e.g., “cell line*” OR “cell culture*” AND “misidentification” OR “authentication” OR “contamination” OR “short tandem repeat*” OR STR OR SNP OR “mycoplasma” AND “cost*” OR “economic”). Additionally, the reference lists of key primary studies and reviews, as well as guidelines and policy documents from major organizations (e.g., ANSI/ATCC, NIH, FDA, EMA, ICLAC, ATCC, DSMZ, Cellosaurus), were screened to capture relevant grey literature. Eligible sources included peer-reviewed articles, guidelines, consensus statements, and reports providing empirical data or qualitative/quantitative estimates on (i) the prevalence or consequences of cell line misidentification or contamination, (ii) performance characteristics of authentication methods (e.g., STR, SNP panels, lpWGS, dPCR, methylation clocks), or (iii) regulatory, ethical, data-stewardship, or economic aspects of authentication; highly technical method papers without a clear link to cell identity or provenance were not prioritized.

### 2.2. Principles, Technological Advances, and Practical Limitations of Short Tandem Repeat Profiling

Short tandem repeat (STR) profiling is based on a fundamental genetic principle. Some loci contain 2–6 polymorphic base pair repeats that mutate at a high enough rate to create significant diversity among individuals, but at a low enough rate to persist through cell culture passages [30]. In the 1990s, early forensic kits focused on nine tetranucleotide loci for human identity testing. This method was later adapted for cell-line research, suggesting that a small number of tetranucleotide repeat sequences provided a high discriminatory power, which is already sufficient to discriminate between most laboratory cell stocks [9,31].

Science, however, rarely stands still. Three technological leaps transformed STR profiling from a forensic tool into a customized cell-culture safeguard [28]. The first advancement was multiplex PCR chemistry, allowing for the simultaneous amplification of variant markers. Initially suggested in the ASN-0002-2011 standard [32], a set of eight autosomal STR markers (CSF1PO, D5S818, D7S820, D13S317, D16S539, TH01, TPOX, and vWA) reduce the probability of identity (POI) for the African American and Caucasian populations to 4.09 × 10^−9^ and 1.02 × 10^−8^, respectively [33]. The 2022 updated version of these guidelines added five additional markers (D3S1358, D8S1179, D18S51, D21S11, and FGA), increasing the POI values to 1.14 × 10^−15^ and 2.97 × 10^−15^, respectively [32].

The second advancement was the introduction of capillary electrophoresis with six-dye detection, which improved the resolution of overlapping size ranges and decreased spectral bleed-through [33]. The third, and perhaps most significant, advancement was the creation of curated reference databases such as ATCC STR [34], DSMZ’s Cell Line Portal [35], and CLASTR at Cellosaurus [36,37]. Additionally, the recently updated Short Tandem Repeat DNA database hosted by the National Institute of Standards and Technology (NIST) provides in-depth information about STRs and other identification markers used in human identification testing [38,39]. These repositories and databases offer allele frequencies, ladder calibrations, and quality control (QC) metrics.

Modern 24-multiplex assay kits, such as ThermoFisher’s GlobalFiler™ (Waltham, MA, USA), now analyze 21 autosomal loci and three sex-determining markers (Amelogenin, DYS391, Y-Indel), significantly increasing the discriminative power of the marker set by several orders of magnitude. The redundancy among sex markers helps to address the rare but documented issue of Amelogenin dropout, which can be caused by Y-chromosome deletions in certain leukemias and other tumor cells [40]. These updated kits also include internal QC fragments, which are synthetic sequences of known length and fluorophore. These fragments can help identify issues such as partial inhibition or instrument malfunction.

However, every strength comes with a limitation. Tumor lines that are highly aneuploid may exhibit allele imbalance or unique peak patterns that present challenges for automated calling algorithms. Allele dropout can occur when DNA quantity falls below 100 pg or when high salt or detergent levels inhibit Taq polymerase [32]. Moreover, stutter peaks, PCR artifacts that are one repeat unit shorter than the true allele, can obscure minor contributors in scenarios involving mixed-cultures. While software solutions like GeneMapper^®^ ID-X [41], STRait Razor [42], and the open-source Haplotype inference and phasing for STRs (HipSTR) [43] include peak-height ratios, heterozygosity thresholds, and ladder alignment checks to minimize false calls, expert review remains crucial [44].

Interpretation standards add another layer of complexity. According to ANSI/ATCC ASN-0002-2022 [32], a “match” requires ≥80% allele concordance across shared loci when 13 core STR loci are compared between two samples. A “partial match” between two samples is defined by an identity of 60–79% and requires further investigation, while anything lower is considered a “mismatch” that requires follow-up [32]. These thresholds were chosen to account for minor drift, such as the loss of the Y chromosome in culture, that is observed in about 45% of male-derived cell lines [45], or the occurrence of extra alleles and microsatellite instability, while still identifying biologically significant divergence [32].

It is important to distinguish between the ANSI/ATCC allele-concordance criteria and the Tanabe similarity coefficient used in some STR matching tools (e.g., CLASTR). ANSI/ATCC ASN-0002-2022 defines a “match”, “partial match”, and “mismatch” based on the percentage of alleles that are concordant across shared loci (≥80%, 60–79%, and <60%, respectively, with at least 13 autosomal loci compared). These thresholds are intended to account for modest genetic drift while still attributing profiles to the same donor. In contrast, the Tanabe similarity score is a mathematically distinct index that quantifies the overlap in allele sets between two profiles. It is often interpreted using more conservative cut-offs (e.g., ≥90% similarity) for declaring “same cell line” in high-stringency applications. In this review, references to the ANSI/ATCC ≥80% concordance rule adhere to the formal standard. Any use of ≥90% Tanabe similarity reflects a stricter, tool-specific criterion. These thresholds are related but not equivalent and should not be confused.

Laboratories seeking accreditation under CLIA or ISO 17025 [46] must conduct positive and negative controls with every batch, document lot numbers, and participate in external proficiency testing. Moreover, introducing such test systems must be critically validated under these guidelines before becoming part of forensic assays [46]. Likewise, participation in studies that compare results from different laboratories is an effective way to identify issues affecting genotyping accuracy, which can be properly addressed in current and future kit systems [47].

Finally, STR profiling is increasingly integrated with barcoding and Laboratory Information Management Systems (LIMS) [48]. For example cryovials, culture flasks, multi-well plates, and any derivative sample containers can be labelled with unique 1-D or 2-D barcodes that are recorded in the LIMS together with the corresponding STR fingerprint. Once an electropherogram is generated, the corresponding allele table is uploaded to the system, encoded into a concise digital identifier, and automatically compared against entries in the local registry. Any deviation beyond predefined limits will prompt a digital non-conformance report. By incorporating molecular identity into the informatics infrastructure, laboratories shift authentication from sporadic “fire drills” to continuous quality assurance. In addition, it will help to establish databases in which the molecular signature of humans can be stored in the form of a barcode. Strategies such as “human identification barcode systems” are discussed in forensic STR profiling for the prevention of crime [49]. However, it should be noted that the establishment of molecular databases of humans bears substantial ethical concerns about consent and misuse.

## 3. Complementary and Emerging Authentication Technologies

While STR profiling remains the undisputed standard in human cell authentication, no single method can address every borderline case. For example, consider a pluripotent stem cell line that undergoes extensive CRISPR editing, resulting in large chromosomal inversions. While STR loci may still match the parental genotype, the functional attributes, and therefore the experimental validity, could be compromised. In these cases, complementary tools are needed to fill the blind spots.

Single-nucleotide-polymorphism (SNP) panels serve as the first tier of augmentation. Commercial kits like Fluidigm’s 96.96 Dynamic Array interrogate a few hundred ancestry-informative markers, providing a level of information comparable to a 24-locus STR while reducing the cost per data point [50]. However, they also offer the added benefit of detecting copy-number variations (CNVs) larger than 50 kb. Because SNPs are bi-allelic, downstream analysis can utilize simple binary matching algorithms, enabling high-throughput screening in core facilities.

Low-pass whole-genome sequencing (lpWGS) has recently reached the significant milestone of costing less than €150 per sample [51]. At 1–2× coverage, lpWGS may not be able to detect most point mutations, but it is highly effective in identifying CNVs, aneuploidy, and species contamination. Importantly, CNV profiles generated by lpWGS can be integrated with single-cell RNA-seq analyses. For example, tools like CONICSmat first infer large-scale copy-number states from the expression matrix and then overlay these states onto the corresponding UMAP/t-SNE plots. This enables researchers to correlate structural variants with gene-expression changes in cell-line sub-clones, organoids or primary tissue samples [52,53].

Digital PCR (dPCR) or digital droplet PCR offers a unique advantage. It requires low DNA quantities and provides exquisite sensitivity for absolute quantification of target nucleic acids [54,55,56]. In workflows involving admixtures, such as patient-derived xenografts where human tumor cells grow in mouse stroma, dPCR can detect human DNA in a murine background [57]. This outperforms electropherogram-based methods, which struggle to detect minor-allele contributions below 10%. Using a two-color probe set that targets human Alu sequences versus murine B1 elements, dPCR can provide a contamination readout in under 60 min.

Methylation barcodes and epigenetic clocks represent the latest advancements in the field [58,59,60]. Even samples with a correct STR profile can be impacted or experience methylation drift [61], This suggests that a fraction of STRs can act as regulators of genome function by modulating DNA methylation, which may impact molecular mechanisms affecting disease or altering drug-response pathways [61]. Bisulfite padlock probes that target age-informative CpG islands can therefore be suggested as an “epigenetic odometer,” allowing for the monitoring of age-related changes independent of genetic information. Though still in the experimental phase, it is reasonable to suggest that integrating STR, SNP, and methylation signatures could offer a holistic understanding of cellular origin and health.

For non-human models, species-specific or custom-designed STR and SNP arrays to identify different breeds are becoming more common [62,63,64]. Canine genetic testing panels of SNP clusters, nonsense variants, frame-shift insertions or deletions (indels), and long STR expansions can distinguish between popular dog breeds, which is important for toxicology studies [65]. In addition, tests have been developed to distinguish between different cell lines from non-human models. For mouse there is a Mouse Universal Genotyping Array (MUGA) available that can be combined with a computational method called Cell Line Authentication by SNP Profiling (CLASP) for cell line authentication and copy number analysis [66]. The robustness, simplicity and curated content of the third generation of this genotyping array (GigaMUGA) scoring for 143,259 probes showed high performance in a set of 500 high-quality reference samples spanning laboratory inbred strains, recombinant inbred lines, outbred stocks, and wild-caught mice [67]. Similarly, a multiplex microsatellite marker panel containing 87 microsatellite markers can be used to authenticate rat strains with only 8 reactions [68]. In addition, commercial providers offer authentication tests for human, dog, mouse, and rats.

More general species identification of cell lines and detection of cross-contamination can also be done by targeting the mitochondrial genomes. Cooper and colleagues implemented a two-pronged molecular approach in which they combined a multiplex PCR-based assay to identify the most common cell culture species and a protocol for sequencing of a 648 bp region located in the cytochrome C oxidase I (COI) gene, i.e., the “barcode region”, that accurately determines the species of cell lines [69].

Plant researchers use simple sequence repeat (SSR) assays, which are suitable for verifying the purity of cultivars in *Arabidopsis* and maize [70]. Therefore, multi-species, multi-omic authentication is no longer just a futuristic idea but a necessary practice in co-culture, microbiome, and ecological research fields.

## 4. Authentication Considerations for Non-Human and Mixed-Species Cultures

Human studies may dominate biomedical headlines, but a significant portion of basic and translational research relies on other organisms. Murine hybridoma libraries provide monoclonal antibodies, insect cells power baculovirus expression systems, and plant protoplasts allow for CRISPR screens in agriculture [71,72,73]. Each system presents unique authentication challenges that the scientific community is only just starting to comprehensively address.

For example, mouse cell lines can be traced back to a small group of inbred founders. Substrains like C57BL/6J and C57BL/6N, which diverged in 1951, and many other mouse strains commonly used in transgenic and knockout production have differences in metabolism, vision, immunopathology, and behavior [74]. A multiplex PCR assay that targets 18 mouse-specific tetranucleotide STR markers (1-1, 1-2, 2-1, 3-2, 4-2, 5-5, 6-4, 6-7, 7-1, 8-1, 11-2, 12-1, 13-1, 15-3, 17-2, 18-3, 19-2, and X-1), as well as two human STR markers (D4S2408, and D8S1106) for contamination detection is widely used for mouse cell line authentication [75]. This panel can distinguish between the identity of mouse cell lines with over 98% confidence [75]. Laboratories conducting behavioral neuroscience studies must, therefore, authenticate not only at the species level but also at the substrain level to avoid any confusion in interpreting phenotypic results.

Hybridomas present a unique challenge as they are intentionally created fusions, typically between mouse myeloma cells and B-cells from various species, including humans [71]. These hybrids can produce multi-allelic STR patterns that complicate standard matching algorithms [76]. ISO working groups are currently developing customized “fusion fingerprints” to document the expected heteroploid alleles. It is also recommended that journals specializing in antibody engineering advise authors to include both parental and hybridoma fingerprints when submitting their work.

Insect cell lines, such as Sf9, High-Five™, and Drosophila S2 allow for high-yield production of recombinant proteins, but they do not have the same extensive reference databases as mammalian systems. Fifteen years ago, several PCR-amplified sequences from selected loci were defined, capable of distinguishing among 40 uncharacterized nucleopolyhedrovirus isolates from larvae of the moth *Spodoptera frugiperda* (commonly known as the fall armyworm) [77]. Until more comprehensive datasets are available, the authenticity of these cell lines must be confirmed through mitochondrial cytochrome c oxidase subunit I (COI) gene sequencing and, when possible, morphological examinations such as observing spindle structure under confocal microscopy [78,79].

Mixed-species systems introduce an additional layer of complexity. Co-culturing human tumor organoids with mouse stromal cells improve physiological relevance but complicates downstream omics analysis. Peaks from the minor species in STR analysis may be obscured by the dominant component, resulting in uncertain contamination thresholds. By employing dual-color droplet digital PCR (dPCR) and low-pass whole-genome sequencing (lpWGS) with species-agnostic bioinformatics tools, like Xenome, which can perform fast, accurate and specific classification of sequence reads [80], it will be further possible to combine genomic and expression data to molecular characterize various cell types, organoids, and tissues. Regulatory agencies such as the U.S. Food and Drug Administration (FDA) mandate explicit documentation of species composition in Investigational New Drug (IND) applications involving xenografts [81].

In agricultural biotechnology, plant cell suspensions like the tobacco Bright Yellow-2 (BY-2) frequently exhibit somaclonal variation, impacting secondary metabolite profiles and reducing productivity [82]. Simple sequence repeats (SSRs) targeting chloroplast microsatellites have been successful for cultivar authentication [83]. However, chloroplast capture and mitochondrial recombination events can lead to inaccuracies with organellar markers. Therefore, nuclear SSRs remain crucial for definitive confirmation.

Consequently, all of these different challenges indicate that authentication must be tailored, not duplicated. Applying human-centric standards to other organisms can result in false security and unnecessary expenses. The next crucial step will involve creating a cross-species authentication ontology that defines minimal marker sets, QC thresholds, and a standardized reporting language that is consistent among scientists working with cells.

## 5. Global Regulatory and Publishing Landscape for Cell-Line Authentication

Policy adoption often lags behind scientific discovery, but in the realm of cell-line authentication the gap is closing rapidly. The first formal guideline, ANSI/ATCC ASN-0002, appeared in 2010 and was updated in 2022 following a two-year public comment period [32]. The revision clarified match thresholds, added requirements for mycoplasma screening, and mandated retention of raw electropherograms for a minimum of five years. Laboratories accredited under Clinical Laboratory Improvement Amendments (CLIA) in the United States or ISO 15189 internationally, must now demonstrate compliance during on-site inspections, including documentation of instrument calibration and analyst competency [84]. This will also require documentation of cell line authentication when cell-based assays are used. Funding agencies have followed suit. The U.S. National Institutes of Health (NIH) issued Notice NOT-OD-15-103 titled “Enhancing reproducibility through rigor and transparency”, compelling grant applicants to include a “rigor and transparency” plan outlining how they will authenticate key biological materials [85]. In Europe, many countries have similar advice for cell authentication in biomedical studies given under “open science and research integrity” requirements, or “Good Scientific Practice” guidelines. For example, the German Cancer Research Center has a mandatory requirement that all manuscripts to be submitted must authenticate the cell lines used in the experiments prior to submission [86]. Similar guidelines for the use of cell lines in biomedical research including cell line development, acquisition and authentication were directed to scientists in the UK [87].

Journals are moving quickly, potentially due to the significant cost of making corrections after publication. The International Journal of Cancer (IJC) has implemented measures to ensure that papers accepted for publication are not based on findings from cross-contaminated or misidentified cells. As a result, authors using established cell lines must provide a certificate of authentication that is not older than three years, confirming the origin and identity of the cells [88]. The Nature Publishing Group “strongly encourages” authentication and reserves the right to delay acceptance until certificates are submitted [89]. Journals from the American Association for Cancer Research (AACR) have automated the process: authors upload a PDF certificate during online submission, which is then verified by an internal tool to confirm the date, locus count, and match status [90]. Additionally, AACR journals request that authors utilize resources such as SciScore [91] or RRID [92] to provide further transparency in their submitted studies. Similarly, journals within Wiley’s group have recently introduced guidelines to prevent the publication of studies involving misidentified or cross-contaminated cells [93].

Regulatory agencies overseeing therapeutics have added additional layers of guidance. The U.S. recommendations of the FDA on human gene therapy products now emphasize precise characterization when cells are used. For example, the “Chemistry, Manufacturing, and Control (CMC) Information for Human Gene Therapy Investigational New Drug Applications (INDs) includes the following statement: “You should identify your cells through tests that distinguish them from other cell lines used in your facility. For cell lines that you have purchased from a type collection, vendor, or received from another investigator, we recommend master cell bank (MCB) testing to confirm the purity of the cells by genetic analysis (i.e., short tandem repeat analysis or other profiling analysis)” [94]. The European Medicines Agency (EMA) also includes similar language in its advanced therapy medicinal product (ATMP) framework, adding cell line authentication to good manufacturing practice (GMP) audits. They state “Where the product contains cells or tissues, a detailed description of these cells or tissues and their specific origin, including the species of animal in cases of non-human origin, shall be provided [95]. The guideline Derivation and Characterization of Cell Substrates Used for Production of Biotechnological/Biological Products also sets landmarks for the characterization of cell-based products [96]. These guidelines emphasize the importance of clear and complete records defining the source, traceability, genealogy, history, identity, purity, and quality of the host cells from which a cell–substrate was generated [96], with STR profiling being an acceptable method. However, some statements in these guidelines such as “*Appropriate testing regimens and test methods for cells used in the production of specific products will vary depending on the donor species used as a source of tissue* ……” highlight the need for proper authentication methods in various areas [96]. In some cases proper methods for authentication are missing, leading to statements like “For continuous cell lines of metazoan origin, it is usually adequate to quantitate culture duration by estimation of either the number of population doublings, the number of subcultivations at a defined dilution ratio, or time in days”. Additionally, China’s National Medical Products Administration (NMPA) has updated and strengthened its “Technical Guidelines for Biological Drug Development”, including cell and gene therapy products to align with the Quality of Biotechnological Products standards [97]. Moreover, proficiency testing programs are expanding, with the College of American Pathologists (CAP) offering a semi-annual Cell Line Identity Survey, also known as Parentage testing. Participating labs receive four blinded samples (two shipments of mother and child specimens), submit STR profiles, and receive z-score feedback [98]. ISO is leading a global inter-laboratory comparison (ILC) that includes molecular, cytogenetic, and phenotypic assays. Participation in these programs is likely to become a de facto requirement for high-impact publications and GMP manufacturing licenses [99].

The regulatory message is clear: authentication has evolved from a best practice to a mandatory standard. Laboratories that postpone compliance not only risk manuscript rejection but also face grant withdrawal, setbacks in product licensing, and, in extreme cases, civil liability. Conversely, early adopters gain a competitive advantage: quicker peer review, streamlined regulatory audits, and increased credibility with investors and collaborators.

## 6. Short Tandem Repeat (STR) Profiling and STR Databases

**Figure 4:**
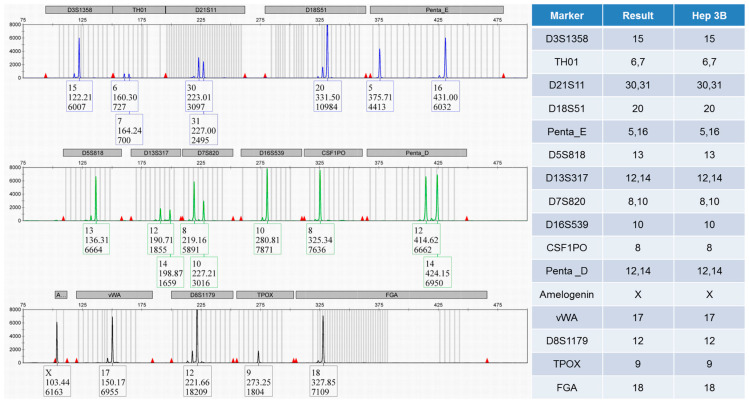
Authentication of the human hepatocellular-carcinoma line Hep 3B by short tandem repeat (STR) analysis. The panel on the left displays the STR profile obtained from the test culture of human liver carcinoma-derived cell line Hep 3B (CVCL_0326). Depicted are the electropherogram peaks and allele calls for the recommended ANSI/ATCC ASN-0002-2022 core loci CSF1PO, D3S1358, D5S818, D7S820, D8S1179, D13S317, D16S539, D18S51, D21S11, FGA, TH01, TPOX, vWA, the sex marker AMEL, and two additional variant markers (Penta D and Penta E). The table on the right compares each STR marker result with the reference profile for Hep 3B [100]. No discordant or missing loci were detected using the Tanabe match algorithm, yielding a calculated similarity score of 100%. According to the ANSI/ATCC interpretive criteria (≥90% similarity = same cell line), these data confirm that the analyzed culture came from the original isolate of the donor and is an authentic Hep 3B line.

One microliter of crude cell lysate is enough to generate a meaningful STR profile in less than half a working day. The read-out, consisting of pairs of allele sizes, can fit on a single line of text, be sent via email as plain ASCII, and be automatically processed by LIMS, manuscript submission portals, or similarity search engines. The simplicity of STR authentication is best understood through practical application: Figure 4 displays a Hep 3B culture with an allele table that matches 100% with the published reference profile, meeting the ANSI/ATCC ≥ 90% criterion with certainty. Producing such a result only requires multiplex PCR and capillary electrophoresis technology that is already available in many standard molecular biology laboratories.

**Figure 5:**
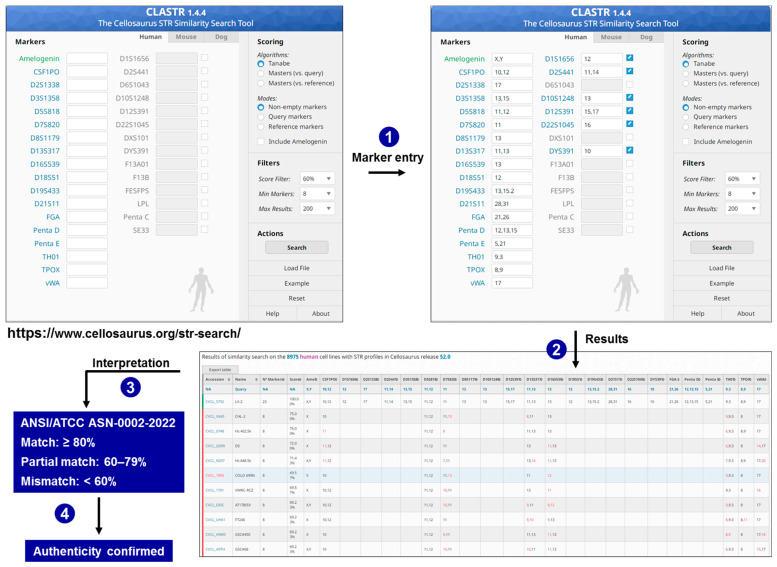
LX-2 cell-line authentication using the CLASTR STR similarity search tool [102]. The figure demonstrates the step-by-step process of using CLASTR to confirm the identity of the human hepatic stellate cell line LX-2. A CLASTR entry mask is displayed immediately after selecting the “STR Similarity Search” option at [102], showing default parameters (species = Human, score filter = 60%, Min Markers = 8; Max. Results = 200) and default scoring parameters (Algorithms = Tanabe, Modes = Non-empty markers). In the current version, entry of 31 different allelic markers and information for the Amelogenin gene for gender determination is possible. When conducting the CLASTR search with 24 markers, the results show a 100% match with the results stored in the Cellosaurus database, where the cell line is listed under CVCL_5792. According to the ANSI/ATCC ASN-0002-2022 guidelines [32], two cells are considered to have been derived from the same donor when they have a match of ≥80%, with at least 13 markers compared. In the CLASTR search differences in markers are highlighted in red.

Researchers have multiple options for matching samples to existing references. The most basic method involves manually comparing the sample with the certificate of analysis (CoA) provided by a biobank or with allele tables found in the literature. A more advanced approach is to input the profile into a spreadsheet or an in-house script that calculates simple concordance or Tanabe similarity against a locally stored reference list. For quick, large-scale searches, web tools like the Cellosaurus STR Similarity Search tool CLASTR [37], ATCC Public STR database [34], or DSMZCellDive [35] can be used. Additionally, the forensic database STRidER contains many human STR genotypes from forensic samples that allow for estimating the probability of identity of finding an STR profile from normal diploid human tissues in different human populations [101]. These tools accept raw allele strings and provide ranked hit lists within seconds. The workflow in these web tools, such as CLASTR, is straightforward (Figure 5).

The allele list is entered into CLASTR’s web form, default parameters accepted or modified, and potential matches are ranked within seconds, confirming the authenticity of the query culture (LX-2 in the example shown). Since the assay is affordable, rapid, and does not require specialized bioinformatics for interpretation, STR profiling has become the common language connecting local laboratory work to global repositories like ATCC, DSMZ, and Cellosaurus. Its simplicity eliminates psychological and financial barriers, making “check first, culture later” a practical default for laboratories of any size. Additionally, many modern LIMS platforms include a matching module that automatically checks new profiles against an institution’s internal registry as soon as the data file is uploaded. Commercial genotyping software that is regularly updated, such as GeneMapper ID-X (ThermoFisher, Waltham, MA, United States), and BioNumerics (Applied Maths N.V. Sint-Martens-Latem, Belgium), offers similar functionality offline, allowing secure comparisons behind firewalls, especially when working with patient-derived or proprietary lines [41,103]. Nevertheless, the Cellosaurus database, with its systematically curated information and more than 9000 deposited STR profiles, currently represents the most reliable and comprehensive resource for accurate cell line identification. CLASTR provides much more usable information than the ATCC STR database or the DSMZ search engine because it warns researchers of misidentified cell lines, provides links to a great deal of relevant references, and further allows calculation of match scores for different types of human cell lines (Tanabe, maters vs. Reference profile, and Masters vs. Query profile).

## 7. Data Stewardship, FAIR Principles, and the Architecture of Trust

The moment a capillary electrophoresis trace is exported from the sequencer, the molecular identity of a culture ceases to be a private laboratory artifact and becomes, potentially, a community resource. This transformation imposes both technical and ethical obligations: files must be stored to remain bit-perfect for years; metadata must be rich enough for a stranger to interpret them; and access rights must reflect the dual imperatives of openness and privacy. The Findable, Accessible, Interoperable, and Reusable (FAIR) framework, which stands for Findable, Accessible, Interoperable, Reusable, provides an actionable roadmap but only if laboratories translate its abstract goals into line-item specifications in their standard operating procedures (SOPs) [104].

The “Findable” pillar begins with globally unique, persistent identifiers. For cell lines these are typically Research Resource Identifiers (RRIDs) [105] or Cellosaurus IDs [37]. For data objects, Digital Object Identifiers (DOIs) minted by repositories such as Zenodo, Figshare, or the European Nucleotide Archive, providing persistent identification of documents across networks [106,107]. A well-formed metadata record embeds both IDs, ensuring that any future user can pivot seamlessly from a paper’s Methods section to the raw electropherograms. The process of automated identifier validation has become increasingly straightforward thanks to services like SciCrunch or CrossRef. These platforms allow researchers to validate and link identifiers through Representational State Transfer (REST)ful Application Programming Interfaces (APIs), which serve as intermediaries that enable different software applications to communicate with each other [108]. In essence, when a researcher makes an API call to these services, they can retrieve information about specific identifiers, such as Digital Object Identifiers (DOIs), and confirm their accuracy or obtain related metadata. However, it is important to note that not all electronic lab notebooks have built-in capabilities for direct integration with these APIs. This means that while the technology exists for seamless validation of identifiers, its implementation may vary depending on the specific tools being used in a laboratory setting. In practice, the bottleneck lies not in technological limitations but rather in discipline. Researchers must be trained to create and manage this metadata at the bench level. If this training occurs only at the time of publication, when memories have faded and notes are scattered across personal laptops, the quality of metadata may suffer significantly.

“Accessibility” is often mistaken for being “free for all,” but regulatory regimes such as the EU General Data Protection Regulation (GDPR) [109] and the California Consumer Privacy Act (CCPA) remind us that raw STR profiles from patient-derived xenografts are considered personal data [110,111]. The solution is to implement tiered access. Public repositories can host de-identified allele tables, which contain genetic information that has been processed to protect individual identities. The de-identification can involve techniques such as pseudonymization, where identifiable information is replaced with pseudonyms, or anonymized, where data is stripped of all identifying details to ensure that individuals cannot be re-identified. These allele tables provide valuable insights for researchers while maintaining privacy. In contrast, raw fragment analysis files (.fsa) contain unprocessed electrophoresis data that are typically more sensitive in nature. These files retain complete information about the genetic sequences and their origins, making them subject to stricter access controls. While de-identified allele tables can be made available in public repositories for broader research use, raw .fsa files are usually kept behind institutional firewalls. Access to these files is managed through controlled access committees or data-use agreements to ensure compliance with ethical standards and regulations. Technologies like the Global Alliance for Genomics and Health (GA4GH) Data Repository Service incorporate token-based authentication layers that facilitate secure access to datasets without discriminately disclosing sensitive information. This means that journals can link to specific datasets while ensuring that only authorized users can view or download the underlying data [112].

“Interoperability” is a common issue among various data sets. Electropherograms are often saved in proprietary binary formats, allele tables in a variety of Excel templates, and metadata in unique jargon. The solution to this problem is the implementation of standards. The Minimum Information About a Cellular Assay (MIACA) and the Minimum Information About a Cellular Assay in Regenerative Medicine (MIACARE) checklists help to standardize field names (e.g., “passage_number” vs. “passageNo”) and establish controlled vocabularies for species, tissue of origin, culture medium, and STR kit version [113,114]. Ontologies from the Open Biological and Biomedical Ontology (OBO) Foundry, such as CLO (Cell Line Ontology) and OBI (Ontology for Biomedical Investigations), populate the drop-down menus in modern LIMS software [115,116,117]. This ensures that when one lab records “DMEM” and another “Dulbecco’s Modified Eagle Medium”, the ontology consolidates both under the same concept identifier.

“Reusability”, the fourth pillar, is the ultimate payoff: data detailed enough to support secondary analyses, machine learning (ML) pipelines, and meta-studies that were never envisioned by the original creators. Achieving this goal requires three extras: provenance logs, usage licenses, and quality tags. Provenance extends beyond “who” and “when” to “how”: the thermal cycler make and model, the polymer composition in the capillary, even the exact ladder lot. Creative Commons licenses, CC-BY for unrestricted reuse or CC-BY-NC for non-commercial restrictions, should be affixed not only to manuscripts but to each discrete data object, clarifying legal status [118,119]. Currently, there are six different license types that enable re-users to distribute, remix, adapt, and build upon published material, as long as attribution is given to the creator [120].

Blockchain technology, often hyped in finance, has quietly found a niche in data integrity [121]. Projects like CellChain store a hash of each STR profile on a public ledger, creating an immutable timestamp that proves the profile existed in a given state on a given date [122]. This mechanism is invaluable in patent litigation and regulatory inspections, where the chain of custody must be incontrovertible. A typical workflow involves computing a SHA-256 digest of the allele table, embedding it in an Ethereum smart contract, and returning a transaction ID stored in the LIMS. If the profile is altered, even by a single nucleotide, the hash mismatch exposes the tampering instantly.

The infrastructural backbone that connects these elements are the different types of available LIMS. Modern LIMS platforms, such as Benchling (San Francisco, CA, USA), LabVantage (Somerset, NJ, USA), and open-source alternatives like openBIS (IT Scientific, Zurich, Switzerland), are no longer just passive data dumps [123]. They now offer API endpoints, role-based permissions, and integration hubs for sequencer run folders. A cell line record can inherit metadata from the initial purchase invoice, add passage history from incubator logs, include STR data directly from the genotyper, and synchronize with external repositories at publication time, all without human copy-and-paste errors.

Implementing FAIR principles from scratch may seem daunting, but incremental adoption is effective. Stage one could involve adding Research Resource Identifiers (RRIDs) to lab notebook entries. Stage two might require selecting a community standard template for STR metadata. By stage three, the laboratory could invest in LIMS connectors and blockchain time-stamping. At each stage, the marginal cost is outweighed by the savings in troubleshooting, compliance audits, and reputational capital. In an era where funders increasingly request data management plans (DMPs) or machine-actionable DMPs (maDMPs) evaluated during peer review, robust data stewardship is no longer just a bureaucratic requirement, it is essential for engaging in cutting-edge science [124].

## 8. Automation, Artificial Intelligence, and the Continuous Authentication Laboratory

If the 20th century was defined by manual benchwork punctuated by occasional quality checks, there is a growing trend in the 21st century towards laboratories that authenticate continuously and autonomously. Recent advancements in robotics, ML, and microfluidics are converging to enhance identity verification at multiple stages of sample handling. For example, while traditional methods such as barcode scanning provide basic tracking capabilities, ML algorithms can analyze patterns in data generated during experiments to identify anomalies or potential errors in real-time. Microfluidics contributes by enabling precise control over small volumes of fluids, allowing for automated processes that can verify sample integrity through continuous monitoring. Together, these technologies work synergistically. Robotics facilitate the physical movement and manipulation of samples, while ML enhances decision-making based on data analysis, and microfluidics allows for efficient sample processing and verification. This integration significantly shrinks the temporal gap between error detection and response from weeks to mere minutes.

A prime example of this evolution is the concept of the “dark lab”, also known as “smart lab”, representing a fully automated facility where human presence is optional [125]. In such environments, every tube, plate, and data packet carries a digital twin, a virtual representation stored in the cloud that mirrors its physical counterpart. This digital twin not only includes information about the sample’s identity but also tracks its history throughout experimentation. By continuously updating this virtual model with real-time data from sensors and instruments, researchers can gain insights into sample conditions and ensure compliance with protocols without direct oversight.

At the hardware level, liquid-handling robots equipped with barcode scanners play a crucial role in linking a physical sample to its digital representation as soon as it exits cryostorage [126]. This process establishes a chain of custody for the sample by capturing its unique identifier and associating it with relevant metadata. Advanced systems, such as HighRes Biosolutions’ modular pods, are equipped with environmental sensors that continuously monitor conditions like temperature, humidity, and CO_2_ concentration. These measurements are recorded at regular intervals and incorporated into the metadata of the sample. By the time an aliquot reaches the thermal cycler for STR amplification, these automated systems have generated structured log files that detail essential information about the sample’s handling history. While these logs may not constitute a “page-length dossier” or provide minute-by-minute updates in real-time, they do offer comprehensive documentation that auditors can review to ensure compliance with protocols.

Microfluidic innovation is driving the next advancement [127]. Companies such as Biomeme and QIAGEN are introducing cartridge-based STR assays where amplification, separation, and detection take place within a credit card-sized chip. The chip has the ability to display analysis results on a smartphone connected to the device via Bluetooth or a USB cable [128]. Integration with electrowetting technology and lab-on-a-chip allows for real-time splitting of reaction droplets, enabling duplicate tests or immediate confirmatory single nucleotide polymorphism (SNP) assays without human involvement [129]. In theory, a laboratory could authenticate every passage of every cell line with little more than a conveyor belt feeding chips into a dock under an imaging hood.

Artificial intelligence (AI) plays a pivotal role in the overall technological framework of genomic research. It processes raw signals generated by various hardware components, such as sequencers and sensors, to make informed decisions and derive valuable genomic insights [130,131]. Traditional peak-calling algorithms rely on fixed thresholds (150 relative fluorescence units (RFUs) for analytical, 50 RFUs for stochastic), but ML classifiers trained on hundreds of thousands of labeled peaks can surpass those rules, especially in borderline cases filled with stutter artifacts or dye pull-up. Convolutional neural networks (CNNs) analyze the entire electropherogram image, not just peak heights, capturing nuances like baseline noise or dye artifacts that are not visible to simple metrics [132,133]. Performance metrics from AIQC STR, an open-source project, indicate a 40% decrease in analyst interventions and a 60% reduction in false positives compared to rule-based QC.

Decision support goes beyond just providing information. Reinforcement learning agents can optimize PCR cycling conditions in real-time by analyzing early cycle fluorescence curves to detect trends indicative of nonspecific amplifications. For example, if the fluorescence data suggest suboptimal annealing temperatures leading to increased background signals, the system can automatically adjust these temperatures in subsequent cycles to enhance specificity and yield [134]. Similarly, feedback loops play a crucial role in bridging wet lab and dry lab processes. They allow for continuous monitoring and adjustment throughout the experiment. If a sample does not pass QC, the LIMS automatically generates a work order to either re-extract DNA or schedule a confirmatory run on a different platform, such as digital PCR (dPCR). This capability creates what can be described as a self-controlled pipeline, which proactively addresses issues by implementing corrective actions based on real-time data, thereby maintaining the integrity and reliability of the results.

Chain-of-custody integrity also benefits. Radio-frequency identification (RFID) tags and computer-vision checkpoints ensure that a sample’s physical trajectory matches its digital itinerary [135]. An unexpected detour, such as a tube left on a benchtop outside the robot’s domain, flags a non-compliance event in the quality-management dashboard. Auditors no longer need to flip through binders of manual logs. Instead, they can scroll through an immutable event stream, color-coded by compliance status.

From a cost perspective, adoption barriers are decreasing. Entry-level pipetting robots now retail for less than €25,000, and open-source frameworks like Opentrons allow for community-contributed protocols for STR premixing [136]. AI peak calling tools are freely available under permissive licenses and can run on commodity GPUs. The real expense lies in change management: rewriting SOPs, re-training staff, and re-calibrating risk assessments. However, early adopters, mostly CROs and biotech startups, report productivity gains that far exceed the initial cost. This will help companies to adapt their authentication workflow onto a robotic pipeline and reduce project kickoff times, allowing scientists to focus on hypothesis generation rather than paperwork.

Because several of the automation and AI applications discussed in this section are emerging technologies, the quantitative improvements cited here should be interpreted as proof-of-concept benchmarks rather than universally generalizable performance guarantees. For example, the reported approximately 40% reduction in analyst interventions and approximately 60% reduction in false positives with AI-assisted STR peak calling reflect performance in specific validation datasets and workflows; these gains will likely vary with kit chemistry, instrument type, and laboratory expertise, and they require independent replication in diverse settings. Likewise, reinforcement-learning-based “smart” PCR thermocyclers and blockchain-anchored provenance systems are currently confined to pilot implementations or early-adopter environments, and their description in this review is intended as a perspective on plausible near-term developments rather than as a depiction of current standard practice. Throughout, these examples are meant to illustrate the potential direction and upper bound of what automation, AI, and distributed ledgers might contribute to continuous authentication, not to imply that such performance or infrastructure is already routine in most laboratories.

Finally, automation enhances reproducibility. When every step is scriptable, deviations can be traced and root-cause analysis becomes more effective. This characteristic aligns with regulatory requirements for data integrity, such as the ALCOA+ framework used to ensure data integrity in life sciences. It includes the original ALCOA principles (Attributable, Legible, Contemporaneous, Original, and Accurate) and additional attributes, which are Complete, Consistent, Enduring, and Available [137] (Table 1). Essentially, the automated, AI-augmented laboratory views cell line authentication not just as a single QC obstacle, but as an ongoing data challenge. This ensures that identity verification keeps pace with the speed of modern experimentation.

## 9. Economics of Authentication: From Laboratory Budgets to National Innovation Policy

Arguments for routine cell line authentication often rely on ethos (i.e., doing the right thing), but administrators and policymakers ultimately base funding decisions on logos (i.e., the financial aspect). To measure this, one must consider three levels: laboratory budgets, institutional risks, and national innovation systems. On a small scale, a laboratory typically spends most of its budget on personnel and supplies, with authentication being a small percentage. For example, currently, companies provide a meaningful STR test, including the electrophoretic profile, typically costing around $100–200, while the consumable costs are about $40 for base line STR genotyping [15,138,139]. If a lab has 25 active lines, authenticating at acquisition and every tenth passage, the annual cost is approximately €4000. While this may seem insignificant compared to other expenses, the benefits can be significant. For instance, identifying a misidentified cell line will save money. In 2021, Korch and Capes-Davis estimated the financial consequences of using the two HeLa contaminated cell lines HEp-2 and Intestine 407. They concluded that roughly $990 million were spent to publish the 8497 HEp-2 and 1397 Intestine 407 studies in which these false cell lines were used [19]. Moreover, further calculations have estimated that the costs resulting from the usage of contaminated or misidentified cells could cost the US over $28 billion/year from which 36.1% are believed to be linked to biological reagents and reference materials [15,140]. Compared to a single batch of high-end antibodies, a two-week Ph.D. salary, or the overall estimated costs resulting from cell misidentification, the estimated costs for STR profiling become almost trivial.

Institutional finances are directly impacted by the scale of the stakes involved. Estimating that a mid-sized research university manages around 400 active biomedical projects, and the institution centrally funds authentication, annual costs could realistically reach about €1 million (€2500 per project). However, the repercussions of a single high-profile retraction could put tens of millions of future grant overheads at risk. Damage to reputation after retracting papers for any reason also plays a crucial role in faculty recruitment and philanthropy efforts [16]. For instance, a Swedish university experienced a series of oncology retractions in 2018, resulting in a significant decrease in competitive funding and early retirement from a scientific career of involved authors [141,142].

National and supranational agencies are currently experiencing macro-economic ripple effects. Irreproducible preclinical studies are leading to inefficiencies in drug development pipelines. It is estimated that the research and development costs for new drug developments are in the range of $161 million to $4.54 billion [143]. These figures have prompted policy interventions. The NIH now requests in grant applications the authentication of key biological and/or chemical resources including cell lines [144]. Similarly, the EU’s Horizon Europe program includes a 5-point scoring system for proposals in which robust QC plans will certainly be taken into account. Similar AI-based calculations can easily be performed for every laboratory by utilizing the STR profiling template provided in Table 2.

Cost–benefit modeling of a single prompt by AI using OpenAI o3 provides quantitative clarity (Table S1).

Based on this simple prompt and setting parameters typical of a middle-sized biomedical laboratory, the expected return on investment (ROI) would be over 3200% in 5 years. This indicates that routine authentication is the logical choice for every laboratory (Table 3).

ROI is also evident in opportunity capture. Companies entering the field of cell-based therapeutics often engage in co-development agreements with pharmaceutical giants. Having authenticity certificates speeds up due diligence, shortens negotiation periods, and speeds up milestone payments. Therefore, the financial rationale for authentication goes beyond cost savings: it also involves value creation. Just as clean, audited, financial records help companies to access capital in corporate finance, accurate cell lineage records provide credibility in the biopharmaceutical market.

While the economic examples presented here suggest that routine STR-based authentication is highly likely to be cost-saving, these calculations should be interpreted as order-of-magnitude estimates rather than precise forecasts and are sensitive to several key assumptions. In particular, the modeled return on investment depends strongly on the assumed annual probability of cell-line misidentification in the absence of testing, the direct and downstream costs attributed to each misidentified line, and the unit cost of STR testing, all of which can vary substantially between laboratories, institutions, and funding systems. Simple sensitivity analyses indicate that even when using more conservative parameter ranges (e.g., lower misidentification probabilities and lower per-incident damage), the net benefit of routine authentication generally remains positive over a multi-year horizon, but the implied ROI can vary by an order of magnitude. The 3200% 5-year ROI reported in Table 3 should therefore be regarded as an illustrative scenario rather than a universal benchmark. To refine these estimates, future research should prioritize (i) multicenter prospective or stepped-wedge implementation studies that compare misidentification events, retractions/corrections, and full economic costs before and after the introduction of mandatory STR (and complementary) testing; (ii) standardized cost-of-error studies that quantify personnel time, consumables, opportunity costs, and funding impacts per misidentified line; and (iii) macro-level modeling that links authentication practices to translational efficiency metrics such as time-to-IND, phase transition probabilities, and overall Research and Development expenditure. Nevertheless, robust authentication serves as both a financial strategy and a scientific ethical practice.

## 10. Ethical, Legal, and Cultural Dimensions: From the Bench to Society

Ethical discourse on cell line integrity often focuses on waste and reproducibility, but the conversation is broader: it includes patient autonomy, intellectual property, environmental impact, and social trust in science [145]. Ethical lapses rarely occur in isolation; they are intertwined with legal liabilities and cultural norms that influence everyday laboratory practices.

Patient-derived material highlights the tension between open science and privacy. A re-identified STR profile could, in theory, be linked back to an individual donor, exposing sensitive health information. GDPR classifies genetic data as a “special category”, requiring explicit informed consent, data minimization, and the right to be forgotten [109]. As a result, researchers must create consent forms that anticipate extensive data sharing while explaining opt-out options.

Intellectual property adds complexity. According to U.S. patent law (35 U.S.C §101), “products of nature” cannot be patented [146]. A good example is the infamous patent dispute over the *BRCA1* and *BRCA2* genes [147,148]. However, artificially engineered cell lines derived from *BRCA1* mutations can in principle be subject to patent protection. In these cases, accurate authentication is crucial in establishing provenance, especially in priority disputes. As discussed, these examples show that blockchain timestamping offers a tamper-proof record that can support inventorship timelines in court.

Environmental ethics play a crucial role in managing waste streams. The process of routine authentication through STR profiling, like any other laboratory test, requires the use of plastics, reagents, and energy, resulting in a carbon footprint [149]. Currently, there exists no universally accepted life-cycle assessment (LCA) for STR profiling. However, a rough theoretical LCA suggests that one complete STR profile contributes roughly 0.1 kg carbon dioxide equivalent (CO_2_e) (Table 4), comparable to the carbon emissions generated by a full blood examination in a hospital setting [150]. When estimating that a typical cell incubator requires between 100 W/h = 2.4 kW/24 h (small incubator) or 750 W = 18 kW/24 h (large incubator), which is equal to 0.72 kg CO_2_e and 5.4 kg CO_2_e according to the assumption made in the calculation, the carbon footprint to establish an STR profile can even be considered small compared to that resulting from meaningless cell incubation.

Additionally, authentication procedures that help prevent failed animal studies indirectly reduce carbon emissions in vivariums and align with the 3Rs (Replacement, Reduction, Refinement) in animal ethics, as proposed by William M. S. Russell and Rex L. Burch in 1959 [151]. Furthermore, inclusion and equitable access are essential considerations. High-end STR equipment may be unaffordable for low-resource settings, exacerbating global disparities in research quality. International collaborations can help address this issue by supporting regional core facilities or sharing mobile genotyping units. Initiatives like Human Heredity and Health in Africa (H3Africa) have shown that with proper logistics and funding, world-class genomic QC for producing high-quality biological samples is achievable in places like Nigeria and Zimbabwe, democratizing both participation and benefits [152,153].

All these ethical and legal factors provide the foundation for technical solutions. Strong authentication practices such as STR profiling enhance respect for donors, protect intellectual property, support environmental stewardship, and build public trust. When reinforced culturally, from graduate education to journal policies, these frameworks elevate authentication from a mere compliance requirement to a shared moral obligation.

## 11. Future Horizons: Toward Real-Time, Multi-Omic, and Cross-Species Authentication

Predicting the future of technology is risky, but multiple research streams are converging to suggest a plausible scenario where cell line authentication is instant, multi-layered, and species agnostic. This trajectory mirrors the progress of DNA sequencing, which has transitioned from lengthy Sanger protocols to handheld nanopore devices in less than two decades.

The first advancement on the horizon is in-line microfluidic STR monitoring [154]. By using fluorescence resonance energy transfer (FRET) probes, microfluids or lab-on-a-chip can conduct assays in a short time, sending allele calls to central hubs or cloud-based dashboards [155]. Such systems are being integrated into commercial and experimental research tools that are fundamentally changing the workflow in science. They allow for automatic stopping of feed pumps and send a crisis alert to lab management if the contaminant allele surpasses a relative fluorescence cut-off. This new approach shifts QC from batch-mode to event-driven, allowing for containment before contamination reaches irreversible levels. Additionally, authentication is moving towards becoming multi-omic. ML strategies are able to combine genomic and transcriptomic profiles to accurately predict tumor type, identify genetic abnormalities, and provide other clinically meaningful information in clinical routine [156]. Integrated dashboards will soon combine STR/SNP identity layers with transcriptomic health indicators, proteomic stress markers, and metabolomic fingerprints. Machine learning (ML) models trained on this data will generate a “cell fidelity score”, condensing vast amounts of information into a simple, visible format understandable to technicians and external auditors. Initial trials using adaptive multi-scale autoencoders on single-cell RNA sequencing data have shown that these self-supervised clustering methods are highly beneficial for downstream analysis, such as cell trajectory [157]. Moreover, AI-based lineage-tracing algorithms appear well-suited to reconstruct the global timeline of HeLa cross-contamination events. Deep phylogenetic models can exploit subtle mutational drifts, accumulated SNPs, copy-number changes, and mitochondrial haplotypes, to order individual cultures along an inferred contamination trajectory. Coupled with time-stamped passage records, Bayesian neural networks can then assign probabilistic dates to each branching event, revealing when and where specific cultures diverged from the original HeLa source. Such data-driven chronologies would complement forensic STR matching and provide laboratories with an objective view of historical contamination chains.

Cross-species barcoding represents the third frontier. High-resolution melting (HRM) assays targeting ultra-conserved elements can distinguish not only between humans and mice but also between numerous mammalian species in a single reaction. In one report, this technology was used to detect cross-adulteration of water buffalo, bovine, goat, sheep, camel, and donkey milks [158]. In the future, this methodology will be crucial for environmental DNA studies and zoonotic pathogen surveillance. A single HRM curve, when paired with ML, can identify the species composition of a co-culture in a very sensitive, cost-effective, and short time, allowing analysis orders of magnitude faster than sequencing.

Regulatory perspectives and frameworks in academia and industry are evolving [159]. In 2014, the FDA established the ‘Emerging Technology Program’ to evaluate innovative technologies, such as AI and ML [159]. Similarly, in 2023 the European Medicines Agency (EMA) shared their perspective on the use of AI and ML in aiding the development, assessment, and monitoring of medicinal products in their “Reflection paper on the use of AI in the medicinal product lifecycle” [160]. While these position papers primarily focus on effective drug development and the use of human and veterinary medicines, they will significantly impact future initiatives promoting “adaptive cell line authentication”. Tong and colleagues introduced a new multi-task framework to recognize cell lines from images and predict the incubation period of cell lines simultaneously. In a pilot study involving data from thirty different cell lines, the methods accurately identified cell lines using deep neural networks on brightfield images with a 99.8% accuracy [161]. It is evident that such tools will be crucial for journals to promptly detect misidentified or contaminated cell lines in submitted papers, when authors do not provide STR profiles for the cell lines used in their research. This advancement will further enhance the integrity of science and make cell authentication as routine as pH measurement, widespread automated, and essential.

It is obvious that the trend of cell line authentication is shifting towards universality, thoroughness, and real-time responsiveness. Future laboratory settings will incorporate microfluidics, embedded sequencing, and AI-driven analytics with secure, cloud-connected provenance ledgers. This integration will establish self-monitoring cell-culture environments where authentication becomes a continuous, seamless function integrated into every experimental process.

## 12. Conclusions, Recommendations, and the Road Ahead

### 12.1. Synthesis of the Evidence

Throughout this manuscript, I have covered fifty years of lessons, from Gartlers’ and Nelson-Rees’ initial warnings to today’s AI-enabled bioreactors. The data-driven narrative is clear. Historical meta-analyses indicate that between 18% and 36% of the world’s most commonly used cell lines are either misidentified or contaminated. Technical studies confirm that modern 24-plex STR kits reduce the likelihood of misidentification to below 10^−16^, while additional assays such as SNP, low-pass WGS and methyl-clock assays further enhance accuracy. Nevertheless, such high numbers of autosomal loci do not provide additional useful genetic information about differences between sublines or a cell line than kits testing for 16–18 loci, but may be suitable for forensic purposes. Economic analyses show that every €1 invested in authentication leads to approximately €6 in savings from preventing downstream costs. Policy evaluations demonstrate a growing global consensus, from ANSI/ATCC to FDA and EMA, that authentication has transitioned from being a best-practice to a mandatory requirement. In summary, scientific, financial, and regulatory factors all point towards the same conclusion: rigorous and routine cell line authentication is now a necessity, not an option, to ensure credible and impactful bioscience.

### 12.2. Action Items for Individual Laboratories

For principal investigators and laboratory managers, the path forward can be outlined as a concrete checklist:

(i) Implement a “gatekeeper” rule: no newly acquired or engineered cell line may enter routine culture until its STR certificate is archived in the LIMS.
(ii) Schedule re-authentication at every tenth passage or equivalent timepoint, whichever comes first.
(iii) Combine STR profiling with monthly mycoplasma screens and quarterly cross-species contamination checks using either dPCR or lpWGS.
(iv) Include FAIR-compliant metadata in each authentication event such as RRID, culture medium, passage number, STR kit version and electropherogram hash, and deposit de-identified allele tables in a trusted public repository.
(v) Ensure that every newcomer, from undergraduate intern to post-doctoral fellow, is trained in both the wet-lab protocol and the ethical rationale, so that authentication competence becomes as ingrained as pipetting technique or biosafety etiquette.

Routinely consult and integrate the curated resources listed in Table 5, ranging from ICLAC and Cellosaurus to RRIDs and PubPeer, so that up-to-date reference data, authenticity warnings, and reporting tools are embedded in everyday workflows. This practice not only prevents the use of misidentified cell lines but also elevates the overall quality, reproducibility, and credibility of biomedical research performed in the laboratory.

### 12.3. Institutional and Funding-Agency Mandates

At the institutional level, economies of scale justify centralization. Core facilities should offer bundled “biospecimen MOTs” (Ministry-of-Transport–style inspections): STR/SNP genotyping, mycoplasma PCR, and basic karyotyping, delivered with a single digital certificate that interfaces directly with university-wide LIMS. Grant-writing offices can streamline compliance by embedding mandatory QC milestones in internal routing forms. Funding agencies, for their part, should continue tying disbursement schedules to demonstrable QC achievements; our modeling indicates that even a 5% hold-back on direct costs is sufficient to secure over 90% compliance within one funding cycle. Journals can amplify the effect by operationalizing automated manuscript screening tools that parse authentication certificates during submission, flagging missing or outdated reports before peer review even begins.

### 12.4. Societal, Ethical and Environmental Pay-Off

The benefits of universal authentication extend beyond laboratories and balance sheets, reaching patients whose biopsies contribute to organoid biobanks, taxpayers who support public research, and ecosystems that benefit from reduced carbon footprints. Ethically, authentication promotes respect for individuals through data privacy and donor consent, beneficence by lowering clinical risks, and justice by providing equal access to reliable reagents. Environmentally, life-cycle analyses demonstrate that correcting just one misidentified, high-throughput screen can offset the CO_2_ emissions of numerous routine STR runs. Socially, transparent QC practices help build public trust during a time when misinformation and reproducibility issues threaten to undermine the social credibility of science.

### 12.5. Outlook: From Episodic QC to Continuous Provenance

Aside from STR, there are numerous other methods that allow for accurate identification of cell types. One effective method is molecular fingerprinting, which uses unique molecular markers or patterns within cells to authenticate specific cell types. Flow cytometry is another commonly used technique that examines physical and chemical characteristics of cells by passing them through a laser beam, allowing researchers to differentiate distinct cell populations based on surface markers. Genetic profiling offers an alternative by analyzing specific genes or genetic sequences to authenticate cells based on their genetic makeup, which is particularly beneficial in stem cell research and regenerative medicine. Immunostaining utilizes antibodies that bind to specific antigens on cell surfaces, aiding in the visualization and identification of specific cell types under a microscope. Single-cell RNA sequencing provides insights into gene expression at the individual cell level, crucial for understanding cellular identity and functionality during authentication processes. Lastly, mass spectrometry can analyze cellular metabolites and proteins, offering a biochemical approach to authenticate cells based on their molecular composition. Together, these techniques significantly contribute to reliable cell authentication across various scientific disciplines. All these methods have strengths and weaknesses (Table 6).

However, STR profiling is still considered the gold standard because its highly polymorphic loci provide unmatched discriminatory power, its protocols and allele-calling rules are globally standardized and backed by extensive reference databases, the technique is rapid, inexpensive and requires only minimal DNA. Decades of use in forensics, clinical diagnostics and cell line repositories have validated its reliability, reproducibility and ability to detect even low-level cross-contamination, making it a uniquely practical and universally accepted first-line test for cell authentication.

Looking ahead, I envision an authentication landscape that is real-time, multi-omic and seamlessly integrated into cyber-physical laboratory infrastructure. Microfluidic inline STR chips will sample bioreactors hourly; AI will fuse genomic, transcriptomic and metabolomic telemetry into a single “cell-health” score; blockchain ledgers will secure immutable provenance across international supply chains. In such a future, the question “Has this line been authenticated?” will fade, not because it is unimportant, but because the answer will be self-evident, embedded transparently in every data packet. Achieving that vision will require the concerted effort of researchers, funders, regulators, publishers and software architects alike. Yet the trajectory is already visible, and the incentives, including scientific integrity, economic efficiency, and ethical responsibility, could hardly be more compelling. The time to complete the transition from episodic QC to continuous provenance is now; the tools are in hand, the standards are in place, and the collective momentum is unmistakable. The future of trustworthy biological research depends on it.

## Figures and Tables

**Table 1:** Principles of the ALCOA+ framework to ensure data integrity, accountability and transparency in research.

Principle	Demand	Explanation/Example
Attributable	Each data point must be traceable to its creator.	The electropherogram file is automatically tagged with the analyst’s user ID and the instrument’s serial number. An audit trail in the LIMS shows who imported, reviewed, or edited the data.
Legible	Data must be both readable and permanent.	Instead of handwritten peak annotations on thermal paper, the lab stores PDF exports of the electropherograms plus the native .fsa files; both can be opened on any standard PC five years from now.
Contemporaneous	Data should be captured at the time of the activity.	The robot finishes the STR run at 14:03 and immediately pushes the raw file plus metadata to the LIMS; timestamps reflect real-time capture, not an end-of-day batch entry.
Original	The data should be the primary record, not a rewrite or a copy.	Analysts work from the raw .fsa files when scoring alleles; spreadsheet summaries are linked back to the raw files, which remain unchanged and stored on a write-protected server.
Accurate	Data must be error-free and truthful.	A ladder control and an allelic size standard are run in the same capillary to verify sizing precision; any off-ladder peak is flagged for re-analysis before the profile is finalized.
Complete	No relevant data should be deleted or omitted.	Besides the test sample, the run folder also contains negative controls, reagent lot numbers, instrument logs, and environmental conditions, ensuring nothing needed for re-analysis is missing.
Consistent	Data must follow a logical sequence, with connected timestamps.	Passage numbers in the culture log, the STR file name, and the LIMS record all show, for example “P17”; time stamps flow chronologically from thaw, culture, DNA extraction, and STR run.
Enduring	Data must exist for the required retention period.	Files are mirrored nightly to an off-site server and written to encrypted tape; the institute’s policy specifies a 10-year retention period for human-cell authentication data.
Available	Data must be accessible for review or audit when needed.	During a grant-renewal audit, the PI retrieves any STR profile in less than two minutes via the LIMS web portal. External reviewers are given read-only, time-limited access tokens.

**Table 2:** Short tandem repeat profiling—DATA collection prompt.

Purpose	The purpose of this questionnaire is to collect all numerical inputs needed to perform a cost–benefit analysis of routine Short Tandem Repeat (STR) profiling for cell-line authentication in your laboratory or facility. By completing the items below, you will enable us to quantify: the total direct cost of STR testing over a defined time horizon, the probability and financial impact of cell-line misidentification if no STR testing is performed, and key economic metrics such as break-even point, return on investment (ROI) and net present value (NPV).
Estimation parameters	Please provide a realistic estimate for each item. If a value is unknown, write “?” so we can address it later. Current number of cell lines in use (baseline). Expected number of new cell lines per year. Planned STR-testing schedule per cell line (e.g., “upon receipt only”, “upon receipt + every 6 months”) ……… Cost per STR test (currency and amount). Internal process cost per test (lab time, overhead; same currency). Annual probability that a cell line is misidentified if NOT STR-tested (%). Estimated financial damage for each misidentified cell line (materials, labor, lost data, repeat experiments, etc.). Additional downstream costs per misidentification (paper retraction, reputation, clinical delay; optional). Planning horizon for the analysis (number of years). Discount/interest rate (%) if a net present value (NPV) is required.

**Table 3:** Economic evaluation of routine STR profiling for cell line authentication: Input assumptions, five-year cost–benefit analysis, and key financial metrics.

**Parameter**			**Value**			
Current cell lines (N0)			10			
New cell lines per year			2			
STR-testing schedule			Upon receipt + every 6 months (= 2 tests/year)			
Cost per STR test			€150 (incl. DNA isolation and shipping costs)			
Internal process cost per test			€0			
Misidentification risk without STR			10% per line/year (which is a reasonable conservative estimate, it can significantly increase with the number of cell lines being cultured and due to stress on technical staff)			
Damage per misidentified line			€100,000 *			
Additional downstream costs			€0			
Planning horizon			5 years			
Discount/interest rate			0%			
**Five** **-year economic evaluation (0% discount rate)**						
**Year**	**Cell Lines ^1^**	**STR Tests**	**STR Cost** **(€/yr)**	**Expected Mis-ID Lines w/o STR**	**Expected Damage w/o STR (€/yr)**	**Net Benefit ^2^ (€/yr)**
1	12	24	3600	1.2	120,000	116,400
2	14	28	4200	1.4	140,000	135,800
3	16	32	4800	1.6	160,000	155,200
4	18	36	5400	1.8	180,000	174,600
5	20	40	6000	2.0	200,000	194,000
Total (5 yr)	—	160	24,000	8.0	800,000	776,000
**Key financial indicators**						
**Indicator**			**Value**			
Avoidable cumulative damage (5 yr)			€800,000			
Total STR expenditure (5 yr)			€24,000			
Net financial benefit			€776,000			
Return on investment (ROI)			3233%			
Break-even point			Year 1 (3600 € vs. 120,000 € potential loss)			

^1^ 10 initial lines + 2 new lines added at the start of each subsequent year; ^2^ Net benefit = (expected damage without STR) − (STR cost). * This is a rough downward assumption. Stern and colleagues calculated that a retracted article accounts for a mean of $392,582 in direct costs (SD $423,256) [16].

**Table 4:** Estimated carbon footprint of a single Short tandem repeat profile analysis.

Emission Source	Typical Input Per Sample	Emission Factor (kg CO_2_e Per Unit)	Resulting CO_2_e Per Sample (kg)	Notes/Main Assumptions
Thermocycler + capillary electrophoresis (electricity)	0.20–0.40 kW/h	0.3 kg CO_2_e kW/h (average grid)	0.06–0.12	30–35 PCR cycles; 30 min CE run
Single-use plastics (tips, tubes, gloves, fragment of capillary array)	8–15 g PP	2–3 kg CO_2_e/kg	0.02–0.04	Based on cradle-to-gate PP LCI data
Molecular-biology reagents (master mix, ladders, polymer)	≈0.5 g	1–3 kg CO_2_e/kg	0.001–0.002	Conservative literature range
Sub-total (direct inputs)	-	-	0.081–0.162	Sum of rows above
Laboratory overhead (HVAC, cold chain, waste treatment)	-	+20–30% of sub-total	+0.02–0.05	Highly lab-specific; added as a factor
Estimated total per STR profile	-	-	≈0.10–0.20 kg CO_2_e	Equivalent to driving ~0.5–1 km in an average car
Carbon emissions of common hospital tests	116 g for full blood examination, equivalent to 0.8 km driving in an average car			[150]

Abbreviations used: CE, capillary electrophoresis; CO_2_e, carbon dioxide equivalent; HVAC, heating, ventilation, and air conditioning; kWh, kilowatt-hour; LCI; life-cycle inventory; PCR, polymerase chain reaction; PP, polypropylene; STR, short tandem repeat.

**Table 5:** Useful online resources for the prevention of the use of misidentified cells.

Resource	Remark	Information Provided/Link
ICLAC Registry of Misidentified Cell Lines	Curated by the International Cell Line Authentication Committee; authoritative, widely cited, and freely accessible; updated several times per year; cross-linked to Cellosaurus entries [24].	List of cell lines shown to be misidentified or cross-contaminated; original (claimed) vs. true identity; evidence and primary literature references; ICLAC unique identifier; date of entry/update. https://iclac.org/databases/cross-contaminations/ (accessed on 4 December 2025).
Cellosaurus	Comprehensive, manually curated knowledgebase (>150,000 cell lines); incorporates contamination notes from ICLAC and other sources; assigns RRIDs; monthly releases; downloadable in multiple formats [37].	Full cell-line dossier: synonyms, species, tissue/disease of origin, sex and morphology, STR profile(s), karyotype, misidentification warnings, recommended culture conditions, patent and publication links, cross-references (e.g., RRID, NCBI BioSample). https://www.cellosaurus.org/ (accessed on 4 December 2025).
CLASTR (Cellosaurus STR Similarity Search Tool)	Companion service to Cellosaurus; accepts user-supplied STR or microsatellite profiles to detect matches; useful for routine authentication or contamination checks [36].	Similarity scores between the submitted STR profile and thousands of reference profiles; ranked list of best-matching cell lines; match statistics and graphical alignment; downloadable reports. https://www.cellosaurus.org/str-search/ (accessed on 4 December 2025). https://www.cellosaurus.org/ (accessed on 4 December 2025).
Research Resource Identifiers (RRID)	Part of the Resource Identification Initiative; supported by major publishers and funding agencies; promotes citation transparency and resource tracking.	Persistent unique identifiers (RRIDs) for cell lines, antibodies, plasmids, organisms, and tools; basic resource metadata, supplier information, cross-links to Cellosaurus and other databases; recommended citation format and usage metrics [105,116]. https://rrid.site/ (accessed on 4 December 2025).
National Library of Medicine—NCBI BioSample	Public repository for metadata about biological samples used in sequencing and other studies; integrates with SRA, GEO, GenBank, etc.; searchable via NCBI interface or API.	BioSample accession numbers for cell-line–derived samples; organism, cell-line name, tissue, disease, passage, sex, and contamination notes when provided; links to associated sequence data and publications [162]. https://www.ncbi.nlm.nih.gov/biosample (accessed on 4 December 2025).
ATCC STR Profile Database & Cell Line Authentication Service	Maintained by the American Type Culture Collection; STR profiles downloadable free of charge; “Cell Line Finder” tool for quick matching; fee-based authentication service available.	Reference STR profiles and certificates of analysis for every ATCC-distributed line; contamination/mycoplasma testing status; recommended culture conditions; provenance and patent info. https://www.atcc.org/search-str-database (accessed on 4 December 2025).
DSMZ Cell Line Database (DSMZ CellDive)	German Collection of Microorganisms and Cell Cultures; human and animal lines; updated continuously; freely accessible.	STR, SNP, and isoenzyme profiles; sex, tissue, morphology; mycoplasma test results; misidentification warnings; recommended medium and handling instructions [35]. https://celldive.dsmz.de/ (accessed on 4 December 2025).
ECACC (European Collection of Authenticated Cell Cultures) Catalogue	Part of Public Health England; major European biobank; provides QC certificates and authentication data.	STR profiles, karyotype summaries, mycoplasma status, culture recommendations, provenance details, available services (e.g., DNA fingerprinting). https://www.culturecollections.org.uk/products/cell-cultures/ (accessed on 4 December 2025).
JCRB Cell Bank	Japanese biorepository; STR or isoenzyme authentication performed on all human lines; English interface available.	STR/isoenzyme profiles, karyotype images, culture conditions, contamination alerts, original vs. donor information, related publications. https://labchem-wako.fujifilm.com/europe/cell_bank/cell_line_list.html (accessed on 4 December 2025).
SciScore™	Automated, AI-based tool integrated into several journal submission systems and PubMed Central; evaluates methods sections for rigor and reproducibility criteria.	Generates an overall “rigor score” and detailed report indicating presence/absence of cell line authentication statements, contamination testing, RRIDs, and other key items (e.g., antibody validation, ethics approvals); provides suggestions and links for missing identifiers [163]. https://sciscore.com/ (accessed on 4 December 2025).
PubPeer/Retraction Watch Database	Community-driven platforms that flag problems in the literature, including cell-line misidentification; complementary early-warning resource.	Public commentary on papers, retraction notices, and editorial expressions of concern; reasons for retraction (e.g., use of misidentified lines); links to original articles and follow-up discussions. https://retractionwatch.com/category/pubpeer-selections/ (accessed on 4 December 2025).

**Table 6:** Summary of strength and weaknesses of cell authentication methods.

Method	Core Principle	Main Strengths	Mann Weaknesses
SNP genotyping panels	Allele-specific PCR or microarray interrogation of tens to thousands of single nucleotide polymorphisms	- Applicable to any species (panel dependent) - Detects intra-species misidentification and subtle drift - Digital readout allows for easy databasing	- Lower power per locus compared to STRs - Requires many loci - Commercial panels can be costly - Interpretation standards are less harmonized than STR
Whole-genome sequencing (WGS)/WES	Deep sequencing of entire genome or exome; bio-informatic matching	- Provides ultimate resolution - Distinguishes subclones, contamination, and genetic drift - Works across species - Generates rich ancillary data such as mutations, CNVs, and pathogens	- High cost and data-analysis burden - Turnaround time is several days to weeks - Requires substantial bio-informatics expertise and reference genomes
DNA barcoding (e.g., COI, 12S rRNA)	PCR and sequencing of conserved mitochondrial (or plastid) marker genes	- Confirms species for a wide taxonomic range - Low cost and simple workflow	- Cannot discriminate between lines from the same species - Mitochondrial heteroplasmy may confound calls
Karyotyping/cytogenetics	G-banding or spectral karyotyping to visualize chromosomes	- Detects large-scale chromosomal abnormalities and cross-species contamination - No need for prior sequence data	- Low resolution for intra-species ID - Labor-intensive, requires metaphase cells and expertise
Fluorescence in situ hybridization (FISH)	Fluorescent probes hybridized to metaphase or interphase chromosomes	- Pinpoints specific chromosomal rearrangements - Confirms interspecies hybrids	- Limited loci per assay - Requires specialized equipment and expertise
Isoenzyme (isozyme) analysis	Electrophoretic mobility patterns of species-specific metabolic enzymes	- Historical, inexpensive, and no DNA required - Distinguishes many species (especially human vs. rodent)	- Low resolution and cannot separate most human lines - Many labs no longer offer the service due to laboriousness
Species-specific PCR/multiplex PCR	PCR primers targeting species-unique genomic regions (e.g., 16S rRNA, Alu)	- Simple, cheap, and rapid (≤4 h) - Useful for routine cross-species contamination checks.	- Only provides yes/no at the species level, no intra-species information - Limited to species for which primers exist
DNA fingerprinting by AFLP/RAPD/RFLP	Genome-wide restriction + PCR or random primer amplification; gel/CE patterns	- Species-agnostic; no prior sequence needed - Detects polymorphisms across the genome	- Reproducibility issues; band-calling subjective - Largely supplanted by STR/SNP methods.
Gene-expression profiling (microarray, RNA-seq)	Transcriptional “signature” compared with reference sets	- Can uncover mis-labeling and functional shifts (lineage, pluripotency) - Same data may serve experimental aims.	- Expression is condition-dependent culture conditions can mask identity - Expensive and bio-informatics intense
HLA typing	PCR-SSP, PCR-SSO or sequencing of HLA loci	- High polymorphism allows for fine discrimination among human donors - Useful when HLA match/mismatch matters (immunology, transplantation).	- Limited to human lines - More laborious than STR; smaller public reference sets
Phenotypic/morphological assessment	Light microscopy (cell shape, growth patterns), immunocytochemistry	- Fast and no specialized reagents required - Good for spotting gross contamination (e.g., fibroblasts in epithelial culture)	- Highly subjective and low specificity - Cannot confirm genetic identity.
Flow Cytometry	Rapid analysis of multiple parameters from individual cells in a fluid stream	- Rapid analysis of multiple parameters - High throughput and quantitative data	- Requires specialized equipment - May not distinguish closely related cell types
Single-Cell RNA Sequencing	Detailed gene expression profiles are obtained at single-cell resolution	- Detailed gene expression profiles at single-cell resolution - Identifies functional states	- Expensive and complex data analysis - Not suitable for routine use
Mass Spectrometry	Biochemical composition profiling, which provides unique signatures based on the mass-to-charge ratio of ions	- Biochemical composition profiling provides unique signatures	- High operational costs
Short tandem repeat (STR) Profiling	PCR amplification of 8–24 highly polymorphic STR loci, fragment-length analysis via capillary electrophoresis	- Gold-standard for human cell lines (ANSI/ATCC standard) - High discriminatory power (sibling-level resolution) - Relatively fast (approximately 1 day) and inexpensive - Large public reference databases available (Cellosaurus, ATCC, DSMZ)	- Human-specific - Limited use for non-human species - Cannot detect intra-line heterogeneity below approximately 5–10% - Requires high-quality DNA - Stutter peaks can complicate interpretation

Abbreviations used: 12S rRNA, 12S ribosomal RNA gene; ANSI, American National Standards Institute; AFLP, amplified fragment length polymorphism; ATCC, American Type Culture Collection; CE, capillary electrophoresis; CNV, copy number variation; COI, cytochrome c Oxidase subunit I; DSMZ, Deutsche Sammlung von Mikroorganismen und Zellkulturen (German Collection of Microorganisms and Cell Cultures); FISH, fluorescence in situ hybridization; G-banding, Giemsa-banding cytogenetics; HLA, human leukocyte antigen; PCR, polymerase chain reaction; PCR-SSP, PCR with sequence-specific primers; PCR-SSO, PCR with sequence-specific oligonucleotides; RAPD, random amplified polymorphic DNA; RFLP, restriction fragment length polymorphism; RNA-seq, RNA sequencing; SNP, single nucleotide polymorphism; STR, short tandem repeat; WES, whole-exome sequencing; WGS, whole-genome sequencing.

## Data Availability

No new data was generated for this work.

## Supplementary Materials

The following supporting information can be downloaded at: https://www.mdpi.com/article/10.3390/medsci14010025/s1, Table S1: Artificial intelligence-based cost–benefit analysis for routine cell line authentication.

## Abbreviations

The following abbreviations are used in this manuscript:

AACR	American Association for Cancer Research
AI	Artificial Intelligence
ALCOA	Attributable, Legible, Contemporaneous, Original, Accurate
ALCOA+	ALCOA plus Complete, Consistent, Enduring, and Available
ANSI	American National Standards Institute
API	Application Programming Interface
ATCC	American Type Culture Collection
ATMP	Advanced Therapy Medicinal Product
BY-2	Bright-Yellow-2
CAP	College of American Pathologists
CC-BY	Creative Commons Attribution licence
CC-BY-NC	Creative Commons Attribution-NonCommercial licence
CCPA	California Consumer Privacy Act
CE	Capillary Electrophoresis
CLO	Cell Line Ontology
CLASTR	Cellosaurus STR Similarity Search Tool
CLIA	Clinical Laboratory Improvement Amendments
CNN	Convolutional Neural Network
CNV	Copy-Number Variation
CO2e	Carbon Dioxide Equivalent
COI	Cytochrome c Oxidase Subunit I gene
dPCR	Digital Polymerase Chain Reaction
DOI	Digital Object Identifier
DSMZ	Deutsche Sammlung von Mikroorganismen und Zellkulturen (German Collection of Microorganisms and Cell Cultures)
EMA	European Medicines Agency
EU	European Union
FAIR	Findable, Accessible, Interoperable, Reusable
FDA	U.S. Food and Drug Administration
FRET	Fluorescence Resonance Energy Transfer
GA4GH	Global Alliance for Genomics and Health
GDPR	General Data Protection Regulation
GMP	Good Manufacturing Practice
H3Africa	Human Heredity and Health in Africa
HRM	High-Resolution Melting
ICLAC	International Cell Line Authentication Committee
IND	Investigational New Drug
ISO	International Organization for Standardization
JCRB	Japanese Collection of Research Bioresources
LCA	Life-Cycle Assessment
LIMS	Laboratory Information Management System
lpWGS	Low-pass Whole-Genome Sequencing
MIACA	Minimum Information About a Cellular Assay
MIACARE	Minimum Information About a Cellular Assay in Regenerative Medicine
ML	Machine Learning
MCB	Master Cell Bank
NIST	National Institute of Standards and Technology
NIH	U.S. National Institutes of Health
NPV	Net Present Value
OBO	Open Biological and Biomedical Ontology
PCR	Polymerase Chain Reaction
POI	Probability of Identity
QC	Quality Control
RFID	Radio-Frequency Identification
RFU	Relative Fluorescence Unit
ROI	Return on Investment
RRID	Research Resource Identifier
SNP	Single-Nucleotide Polymorphism
SSR	Simple Sequence Repeat
STR	Short Tandem Repeat
t-SNE	t-Distributed Stochastic Neighbor Embedding
TLA	Three-Letter Acronym
UMAP	Uniform Manifold Approximation and Projection
WES	Whole-Exome Sequencing
WGS	Whole-Genome Sequencing
