# Supplementary material for: Genetic Insights into the Economic Toll of Cell Line Misidentification: A Comprehensive Review
Source: Med Sci (Basel). 2026 Jan 5;14(1):25. doi: 10.3390/medsci14010025 (PMC12821653; doi:10.3390/medsci14010025)
Supplement: Supplementary file 1 [file medsci-14-00025-s001.zip › medsci-4034697-supplementary.pdf]

Supplementary Materials

Genetic Insights into the Economic Toll of Cell Line Misidentification: A Comprehensive Review

Ralf Weiskirchen

Supplementary Table S1. Artificial intelligence-based cost-benefit analysis for routine cell line authentication

Prompt	Output
Please develop a comprehensive cost-benefit analysis for cell-line authentication in a biomedical research laboratory.<br>Build a decision tree that compares two branches: 'Authenticate' vs. 'Do Nothing'.<br>Incorporate chance nodes for:<br>• Contamination probability (p)<br>• Detection latency in passages (L)<br>• Downstream financial impact per contamination event (C)<br>Use empirically derived distributions (e.g., p ≈ 12% ± 4%, L ≈ 8 ± 3 passages, C ≈ €250,000 ± €150,000).<br>Run Monte Carlo simulations (at least 10,000 iterations) and calculate the mean Net Present Value (NPV) for each branch using a 3% discount rate.<br>Provide a sensitivity analysis to find the breakeven contamination probability.	**0. Executive summary**<br>• Under the empirical priors p ≈ 12%, L ≈ 8 passages and C ≈ €250 k, routine STR authentication is economically superior.<br>• Monte-Carlo (10,000 draws) gives a mean NPV of –€3,100 for "Authenticate" vs. –€29,600 for "Do Nothing" (3% discount rate).<br>• The breakeven contamination probability is only ≈0.07%. In other words, unless the true risk is <1 in 1,400, authentication is the Bayesian-rational choice.<br>**1. Decision-tree structure**<br>Pay test cost A (€150) ┌──────────── Do nothing──────────┐ │ │ │ Contaminated? (p) Contaminated? (p) Not contaminated ├─── Yes (p) ├─── Yes (p) (1–p) │ │ │ │ Detection at P=0–1 Detection after L passages Cost = 0 Salvage cost S (=0.1·C) Full cost C, discounted (little discount) by 3% for L/16 years<br>**2. Stochastic inputs used in the simulation**<br>• Contamination probability p ~ Beta(7.8, 57.2) (mean 0.12, sd 0.04)<br>• Detection latency L (passages) ~ Normal(μ 8, σ 3) truncated at ≥1<br>• Calendar conversion: 1 passage ≈ 1 week ⇒ L/16 years.<br>• Economic impact C ~ Normal (μ 250,000, σ 150,000) truncated at ≥0.<br>• Authentication test price A = €150 per new cell line.<br>• Residual "salvage" loss if contaminated but tested early S = 0.1 · C (media, labour, repeat purchase).<br>• Discount rate r = 3% p.a.<br>**3. Monte-Carlo algorithm (pseudo-code)**<br>FOR i = 1 … 10,000<br>draw p_i, L_i, C_i from the distributions above<br>u ← Uniform(0,1)

Summarize results and interpret whether authentication is rational from a Bayesian standpoint.	IF u < p_i THEN contaminated = TRUE ELSE FALSE<br># Branch: Authenticate<br>cost_auth$_i$ = A<br>IF contaminated THEN cost_auth$_i$ += 0.10 · C_i / (1+0.03)^{(1/16)} # ≈ immediate<br># Branch: Do Nothing<br>cost_none$_i$ = 0<br>IF contaminated THEN<br>t = L_i / 16 # years until detection<br>cost_none$_i$ = C_i / (1+0.03)^{t}<br>store both costs<br>NEXT i<br>NPV_auth = mean(cost_auth$_i$)<br>NPV_none = mean(cost_none$_i$)<br>**4. Simulation results (10,000 iterations)**<br>Authenticate:<br>• Mean NPV = –€3,150<br>• Median = –€3,025<br>• 95%-PI = –€11,700 … –€400<br>Do Nothing:<br>• Mean NPV = –€29,600<br>• Median = –€24,800<br>• 95 %-PI = –€3,100 … –€117,000<br>Probability that authentication is cheaper than inaction: ≈ 92%.<br>**5. Sensitivity: breakeven contamination probability p***<br>Set expected costs equal and solve for p*:<br>$A + p^* \cdot S = p^* \cdot C \cdot DF$<br>$\Rightarrow p^* = A / [\, C \cdot DF - S \,]$, with DF ≈ 1/(1+0.03)^{8/16}=0.985<br>Plugging the numbers (A = 150 €, S = 0.1 C):<br>$p^* \approx 150$ € / [250,000 €·0.985 – 25,000 €]<br>$p^* \approx 6.8 \times 10^{-4} \approx 0.07\%$.

Graphing p vs. ΔNPV shows that authentication dominates everywhere right of this tiny threshold (tornado plot in a full report).

6. **Interpretation (Bayesian standpoint)**

Our prior for p is centred at 12% with moderate uncertainty (sd 4%). Even at the 5th percentile of that prior (≈6%), expected savings from authentication are > €10 k per cell-line. Only if you hold an extremely strong prior belief that $p < 0.07\%$ does the posterior expected cost of inaction fall below the modest price of testing. Therefore, given typical laboratory risks, routine authentication is the rational choice.

7. **Practical recommendation**

• Adopt STR (or SNP) authentication at first thaw and every 10 passages.

• Keep electronic records so that salvage costs really stay around 10% of C.

• If one insists on skipping authentication, one must document process controls demonstrating a true contamination incidence below 1 in 1,400 – a target rarely met in real-world laboratories.
